# Use of an endoscopic flexible grasper as a traction tool for excision of polyps: preclinical trial

**DOI:** 10.1038/s41598-021-98162-x

**Published:** 2021-09-21

**Authors:** Shinya Urakawa, Teijiro Hirashita, Yuka Hirashita, Lea Lowenfeld, Krishna C. Gurram, Makoto Nishimura, Jeffrey W. Milsom

**Affiliations:** 1 Department of Surgery, Weill Cornell Medicine/ New York Presbyterian Hospital, 525 East 68th Street, K-801, New York, NY 10065 USA; 2 Department of Gastroenterology and Hepatology, The Brooklyn Hospital Center, New York, NY USA; 3 Gastroenterology, Hepatology and Nutrition Service, Memorial Sloan Kettering Cancer Center, New York, NY USA

**Keywords:** Gastroenterology, Medical research, Oncology

## Abstract

Endoscopic submucosal dissection (ESD) is challenging in the right colon. Traction devices can make it technically easier. In this study, we evaluated a flexible grasper with articulating tip and elbow-like bending (IgE) through a double-balloon surgical platform (DESP), compared with an earlier generation grasper without elbow-like bending (Ig). The reach of Ig/IgE was investigated at eight locations using a synthetic colon within a 3D model. Using a fresh porcine colorectum, 4 cm pseudo-polyps were created at the posterior wall of the ascending colon. Fifty-four ESD procedures were performed using three techniques: standard ESD (STD), ESD using Ig (DESP + Ig), and ESD using IgE (DESP + IgE). IgE was able to reach the full circumference at all the locations, whereas the medial walls proximal to the descending colon were out of Ig’s reach. Compared with the STD, both DESP + Ig and DESP + IgE showed significantly shorter procedure time (STD vs. DESP + Ig vs. DESP + IgE = median 48.9 min vs. 38.6 vs. 29.9) and fewer injuries (1.5 vs. 0 vs. 0). Moreover, the DESP + IgE had a shorter procedure time than the DESP + Ig (p = 0.0025). The IgE with DESP increased instrument reach compared to Ig, and likely represented a traction tool for excision of large pseudo-polyps in the right colon.

## Introduction

Endoscopic submucosal dissection (ESD) has been established as an acceptable technique to treat premalignant colorectal lesions^1–3^.On the one hand, it avoids the morbidity of a colectomy, and on the other hand, it achieves a higher en bloc resection compared to endoscopic mucosal resection (EMR), facilitating accurate pathological diagnosis, curative resection, and reducing the risk of local recurrence^4–6^. However, ESD has not been adopted widely due to technical difficulties, longer procedure times, higher complication rates, and longer learning curves, especially for lesions located in the right colon^7–9^. As a result, more than 25,000 colectomies for non-malignant polyps are performed annually in the United States^10^.

A traction device can increase the efficiency and safety of ESD procedures by maintaining visualization of the submucosal plane^11^. Various traction devices have been proposed to reduce procedure times and complication rates^12,13^. However, most devices are limited in their ability to navigate to the right colon or maintain dexterity of the instruments once situated in the right colon^14^.

A double-balloon endolumenal surgical platform (DESP, Dilumen™ C^2^, Lumendi LLC, Westport CT USA) is one example of a platform used to advance the endoscope to the lesion, improve stability of the colon, and maintain visualization of the lumen throughout the colon^15^. The endoluminal platform also allows the use of dedicated instruments (e.g. grasper, scissors, and knife) through two external tool channels. The endolumenal interventional grasper with an articulating distal tip (DiLumen Ig™, Lumendi) provides a good traction during endoluminal procedures^16^, however, Ig is not necessarily able to reach the lesion or provide traction in all directions due to the limitation of its movement in clinical cases. To resolve these issues, the endolumenal interventional grasper with Elbow (DiLumen IgE™, Lumendi) adds an elbow-like bending section more proximally. In this study, we evaluated the efficacy of a novel flexible grasper with an articulating distal tip and elbow-like bending section (IgE) in a preclinical model, compared with Ig.

## Materials and methods

### Double-balloon Endolumenal Surgical platform (DESP), Endolumenal Interventional Grasper (Ig), and Endolumenal Interventional Grasper with Elbow (IgE)

**Figure 1:**
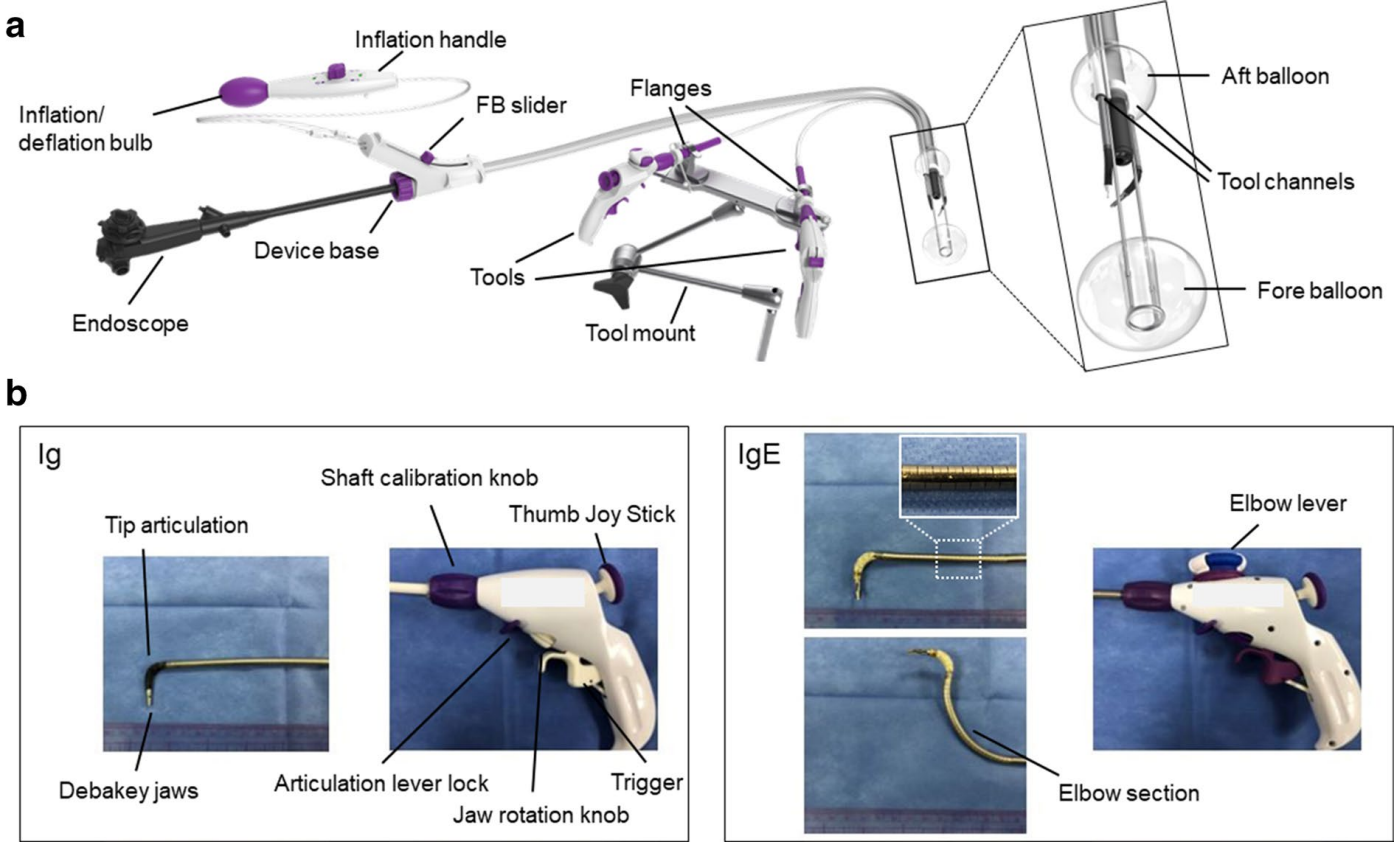
Traction devices. (**a**) Double-balloon Endolumenal Surgical platform, DESP. (**b**) Endolumenal Interventional Grasper, Ig/ Endolumenal Interventional Grasper with Elbow, IgE.

DESP, Ig, and IgE have been approved by the U.S. Food and Drug Administration. The DESP consists of a flexible oversleeve (103 cm, 130 cm) with two independently inflatable balloons and bilateral external tool channels (Fig. 1a)^16^. The Aft Balloon (AB), which is fixed behind the endoscope tip, provides stability for the endoscope. A “therapeutic zone (TZ)” is created by extending the Fore Balloon (FB) beyond the endoscope tip. This adjustable FB improves luminal visualization by flattening folds and straightening flexures. Both balloons are inflated by a pump on the inflation handle^15,16^. Ig and IgE instruments consist of a pistol style handle and flexible shaft with an articulating jaw at the distal tip (Fig. 1b). Both Ig and IgE can be inserted through the external tool channels of the DESP, and the tip can be articulated toward the target using a thumb joy stick. The jaw is closed/opened by squeezing/releasing the trigger and oriented to the desired plane by rotating the jaw rotation knob. The tool channel ports can be secured to the tool mount for additional stability. IgE adds an elbow section, which can bend towards the black line on the shaft by turning the elbow knob (Fig. 1b, Supplementary Video. S1).

### Reach of Ig/IgE

**Figure 2:**
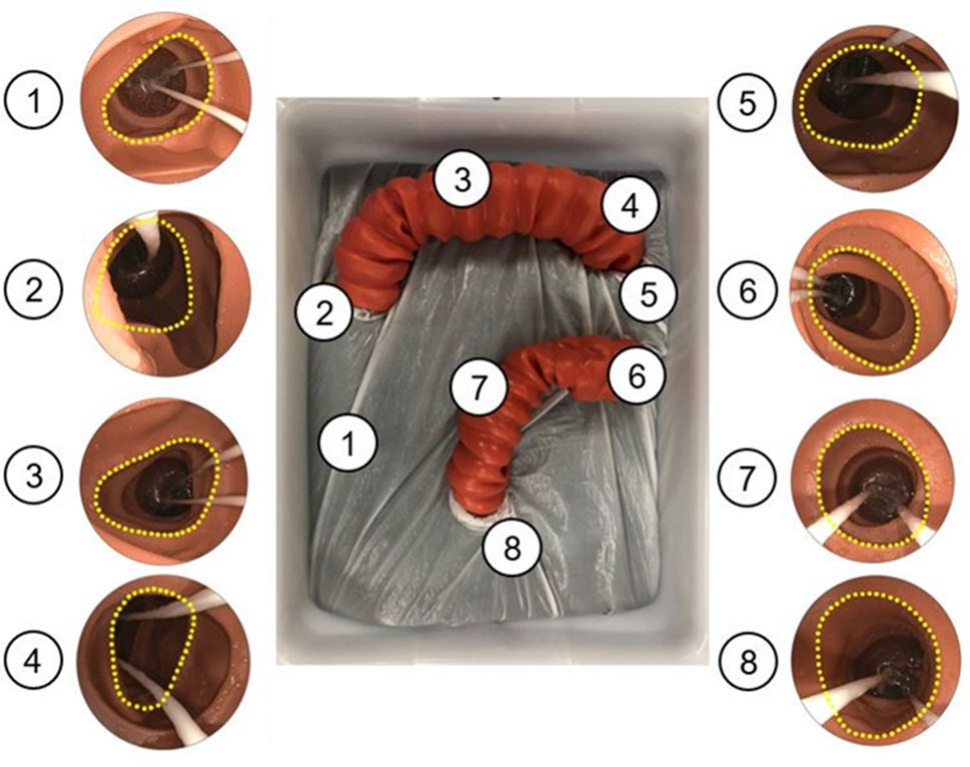
Ex vivo study setting to investigate the reach of Ig/ IgE. The synthetic colorectum was positioned within an established 3D model and fixed by clamps at 4 points. Endoscopic view of Ig/IgE was demonstrated at each position: the ascending colon (site 1), proximal to the hepatic flexure (2), the transverse colon (3), proximal to the splenic flexure (4), the descending colon (5), proximal to the sigmoid-descending colon (6), the sigmoid colon (7), and the rectum (8).

The colonoscope (PCF-H180AL, Olympus), equipped with DESP, was navigated through a 3-D synthetic colon model (Colon-rectum tube, Kyoto Kagaku America Inc, Torrance CA USA) (Fig. 2). Eight target points were selected to evaluate the reach of the Ig and IgE instruments; the ascending colon (site 1), proximal to the hepatic flexure (2), the transverse colon (3), proximal to the splenic flexure (4), the descending colon (5), proximal to the sigmoid-descending colon (6), the sigmoid colon (7), and the rectum (8). At each point, the FB was deployed to straighten the colon and create the TZ. The reach of Ig/IgE, the area where their forceps were able to touch, was investigated when the synthetic colon was distended to its maximum using the endoscope insufflation.

### Ex-vivo setting

**Figure 3:**
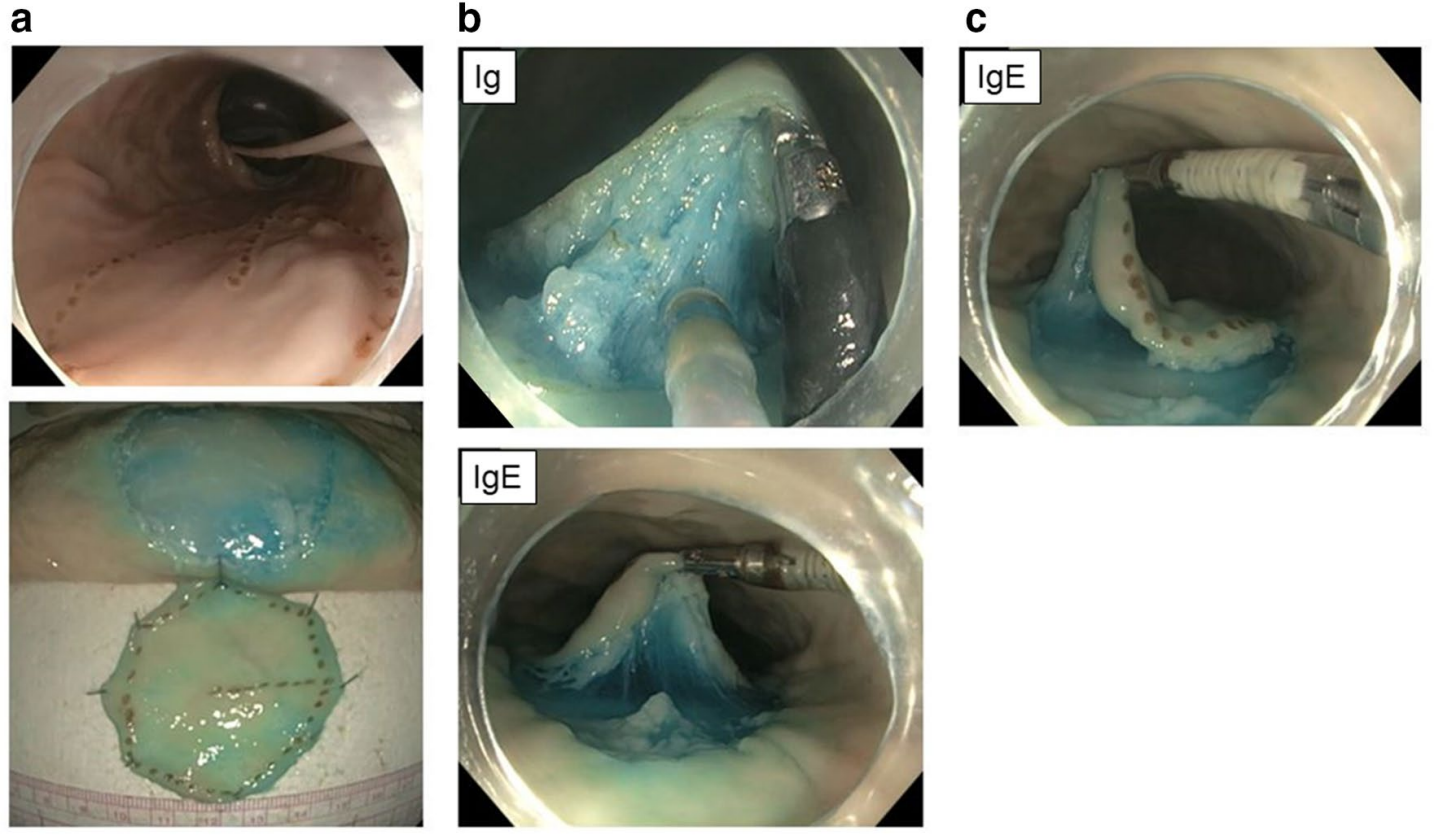
Ex vivo study setting of ESD procedures. (**a**) 4 cm lesions were located at the posterior wall of the ascending colon (upper). Resected specimen with a 5 mm margin (lower). (**b**) Traction provided by Ig (left)/ IgE (right). (**c**) The left edge of the lesion was regrasped by IgE.

In this study, we used ex-vivo porcine colons and no live animals were used. Using electrocautery, 4 cm pseudo-polyp was created in a fresh porcine colorectum and the intestine was positioned within an established 3D colon model (Fig. 3a). The colorectum was fixed by clamps at 4 points, mimicking anatomic retroperitoneal fixation^16^. The pseudo-polyp was placed on the posterior wall of the ascending colon. Three different ESD techniques were evaluated and compared:

1. Standard Cap-assisted ESD technique (STD group)
2. ESD with DESP and Ig (DESP + Ig group)
3. ESD with DESP and IgE (DESP + IgE group)

Six endoscopists who have used this surgical platform several times performed the ESD procedures by these three techniques in a sequential alternating fashion. Clinically, two endoscopists perform colorectal ESD (> 50) and one endoscopist performs EMR (> 100 procedures); these endoscopists were defined as the experienced group. The other three endoscopists only had ESD experience in ex-vivo models (> 10) and were defined as the novice group.

### Navigation to the cecum

A straight transparent cap (D-201–12,704, Olympus) was attached to a pediatric colonoscope. The colonoscope, equipped with/without DESP, was advanced to the cecum. Abdominal pressure and use of the balloon technique were applied to reduce loops and shorten the colon at the endoscopist’s discretion.

### ESD procedures using Ig/IgE with DESP

After navigation to the cecum, the FB and AB were deployed proximal and distal to the lesion. The FB was docked during the procedure at the endoscopist’s discretion. Normal saline with 0.04% methylene blue was used for submucosal injection by injection needle (23G Interject™, Boston Scientific). The same settings (Endo Cut Q mode) of the ERBE electrosurgical unit (Erbe VIO 300D, Erbe USA) was used for all parts of the ESD procedures. Using a DualKnife (KD-650U, Olympus), a circumferential mucosal incision was made around the lesion with a 5 mm margin (Fig. 3a). Once the circumferential incision was completed, the grasping instrument, Ig or IgE, was inserted through the external channel. The Ig or IgE grasped the distal edge of the lesion, retracting it to provide tension to visualize the appropriate plane of dissection (Fig. 3b). Manipulating the tissue in all directions and regrasping it as needed provided traction until submucosal dissection was completed (Fig. 3c, Supplementary Video. S2).

### Outcome measurements

The primary outcomes were the reach of the Ig/IgE and the total procedure time for ESD procedures. Total procedure time was defined as the time from submucosal injection to the removal of the specimen. Secondary outcomes included the cecal intubation time, resected specimen size, grasping time (for DESP + Ig and DESP + IgE groups), submucosal dissection time/speed, the number of muscularis propria injuries, and occurrence of perforations. Submucosal dissection speed was calculated as follows: (major radius) x (minor radius) π/ (dissection time). Grasping time was defined as the time from Ig/IgE insertion through the tool channel to grasping of the lesion.

### Statistical analysis

Sample-size calculation of ESD procedures in this study was as follows; at least 16 procedures were required to detect a decrease in total procedure time from 42 min in the Ig group to 35 min in the IgE group, with 80% power to show a significant difference between groups and 5% type I error. Each endoscopist performed three ESD procedures using each technique.

All variables are expressed as a median. The associations between continuous parameters were evaluated by the Mann–Whitney U test to compare two groups and by the Kruskal–Wallis test to compare more than two groups. Variables with p value < 0.05 in the Kruskal–Wallis test were subjected to multiple comparisons using the Steel–Dwass method. All analyses were performed using JMP Pro 14 statistical DiscoveryTM (SAS Institute Inc., Cary NC, USA). *p* values < 0.05 were considered to be significant.

## Results

### Reach of Ig/IgE

**Figure 4:**
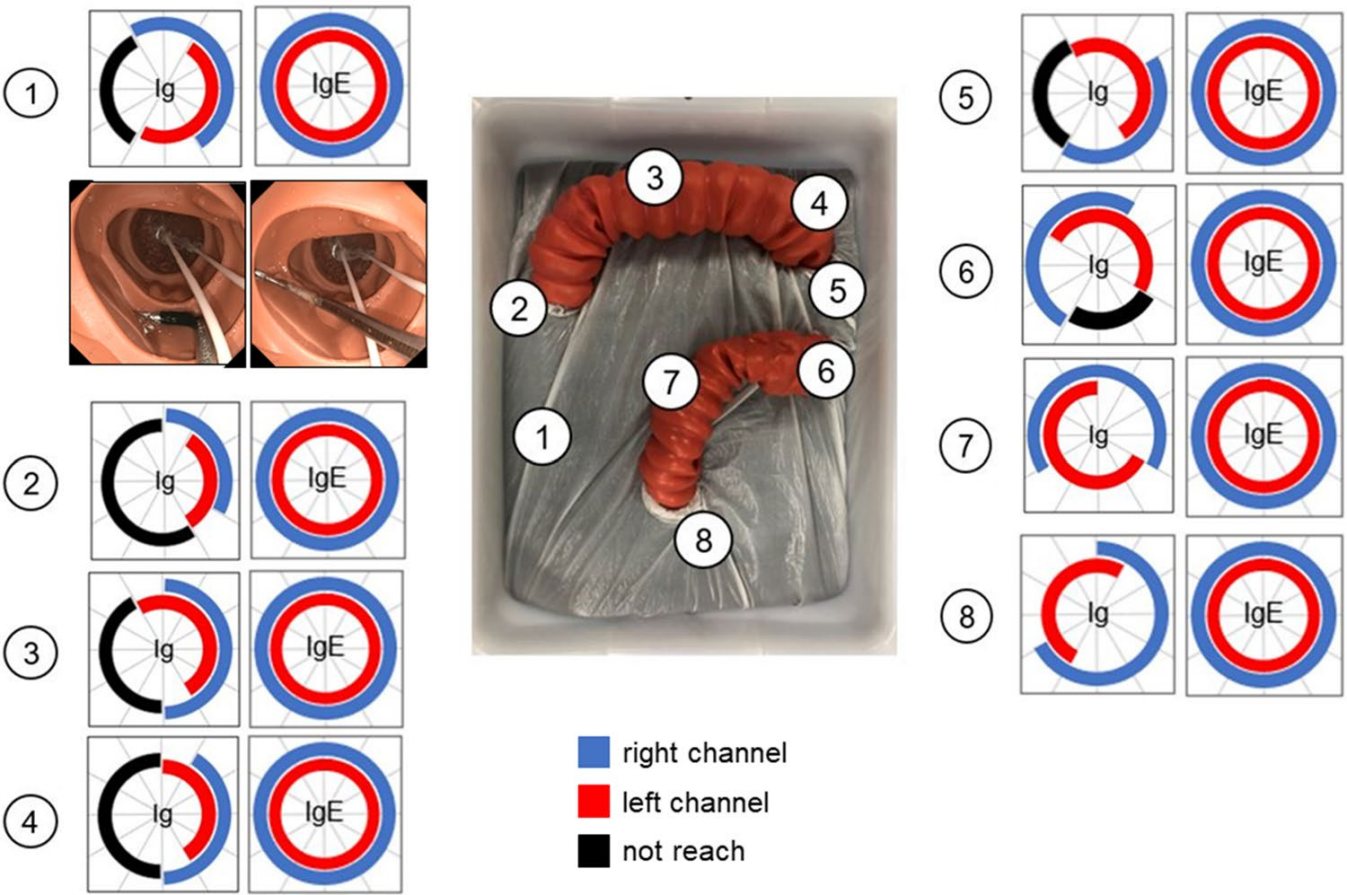
Reach of Ig/IgE. The reach of Ig/IgE was demonstrated at each position (sites 1–8). The range in blue indicates the reach when Ig/ IgE was inserted through the right channel, the range in red indicates the reach when Ig/IgE was inserted through the left channel, and the range in black indicates the area that was inaccessible by either channel. In site 1, a representative endoscopic image shows that IgE was able to reach the area on the left where Ig could not.

Ig was able to reach the full circumference of the synthetic colon at the rectum and the sigmoid colon via both the right and left channels (Fig. 4). However, the posterior wall proximal to the sigmoid-descending colon and the medial wall at the other five locations, especially in the locations immediately proximal to the splenic flexure and the hepatic flexure, were out of Ig’s reach. On the contrary, IgE was able to reach the full circumference at all eight locations by using the elbow bending section, regardless of which external channel was used.

### Navigation to the cecum with/without DESP

A total of 36 colonoscopies were performed (18 without DESP and 18 with DESP). The cecal intubation rate was 100% with no perforation, regardless of the use of DESP. There was no difference in the navigation time (without DESP vs. with DESP = 2.8 [range 1.9–4.7] vs. 2.8 [1.6–3.9] min, *p* = 0.60, Table. 1). Colonoscopies performed without DESP did not require abdominal pressure; abdominal pressure to the left lower quadrant (11/18 procedures, 61.1%) and use of the balloon technique (9/18 procedures, 50%) were applied during colonoscopies performed with DESP.

**Table 1:** Navigation to the Cecum with and without DESP.

	without DESP (n = 18)	with DESP (n = 18)	p
Intubation rate	18/ 18, 100%	18/ 18 ,100%	1.00
Navigation time (min)	2.8 (1.9–4.7)	2.8 (1.6–3.9)	0.60
Abdominal pressure yes/ no	0/ 18	11/ 7	** < 0.0001**
Balloon technique yes/ no	N/ A	9/ 9	N/ A

Median value (range). Bold p values are statistically significant (p < 0.05).

DESP: double-balloon endolumenal surgical platform.

### ESD procedures of 4 cm lesions at the posterior wall of the ascending

A total of 54 ex-vivo ESD procedures were performed with three different techniques (STD (n = 18), DESP + Ig (n = 18), and DESP + IgE (n = 18)). All lesions were successfully resected *en bloc* without perforations. There was no difference in the resected specimen size. Total procedure time was significantly reduced in the DESP + Ig and DESP + IgE groups, compared to the STD group (STD vs. DESP + Ig vs. DESP + IgE = 48.9 vs. 38.6 vs. 29.9 min); this reduction was driven by significantly reduced submucosal dissection time (STD vs. DESP + Ig vs. DESP + IgE = 31.9 vs. 22.9 vs. 16.1 min, Table. 2). As expected, the shorter procedures in the DESP + Ig and DESP + IgE groups used a smaller volume of submucosal injection fluid. Moreover, DESP + Ig and DESP + IgE groups created fewer injuries to the muscularis propria (STD vs. DESP + Ig vs. DESP + IgE = 1.5 [range 0–3] vs. 0 [0–1] vs. 0 [0–2]).

**Table 2:** ESD procedural outcomes at the Ascending colon using STD, DESP + Ig, and DESP + IgE.

	STD (n = 18)	DESP + Ig (n = 18)	DESP + IgE (n = 18)
Resected specimen size* (mm)	53 (51–56)	52 (50–56)	53 (50–61)
Grasping time† (min)	N/A	1.5 (0.77–3.7)	1.9 (1.0–4.5)
Number of re-grasping	N/A	2 (0–5)	1 |||| (0–3)
Submucosal injection volume (ml)	55 (38–62)	38 §§ (32–47)	32.5 §§ |||| (25–52)
Submucosal dissection time (min)	31.9 (22.9–46.2)	22.9 §§ (7.9–39.4)	16.1 §§ || (5.1–29.2)
Dissection speed‡ (cm^2^/hr)	26.6 (18.1–35.9)	56.5 §§ (31.1–150)	78.2 §§ || (48.8–255)
Total procedure time (min)	48.9 (37.8–68.9)	38.6 §§ (21.0–59.9)	29.9 §§ || (17.6–46.0)
Number of muscular injuries	1.5 (0–3)	0 §§ (0–1)	0 §§ (0–2)

Median value (range).

STD: standard ESD technique, DESP: double-balloon endolumenal surgical platform, Ig: interventional grasper, IgE: Ig with elbow.

* The longest diameter of resected specimen.

^†^ Time from Ig insertion through the tool channel to grasping the lesion.

^‡^ (major radius) x (minor radius) π/ (dissection time).

^§^ vs STD, p < 0.05, §§ vs STD, p < 0.01, || vs Ig, p < 0.05, |||| vs Ig, p < 0.01.

Regarding the difference between DESP + Ig and DESP + IgE groups, the DESP + IgE group showed significantly shorter total procedure times (*p* = 0.025), shorter submucosal dissection times (*p* = 0.022), and smaller volumes of submucosal injection fluid (*p* = 0.0069) (Table. 2). For all procedures, the distal edge of lesions was grasped, and there was no difference in grasping time between the two groups (DESP + Ig vs. DESP + IgE = 1.5 vs. 1.9 min, *p* = 0.33). However, the IgE showed a smaller number of times the lesion was re-grasped compared to Ig (DESP + Ig vs. DESP + IgE = 2 [range 0–5] vs. 1 [0–3], *p* = 0.0015).

### Difference in ESD outcomes using DESP + IgE between the experienced and the novice endoscopists

**Figure 5:**
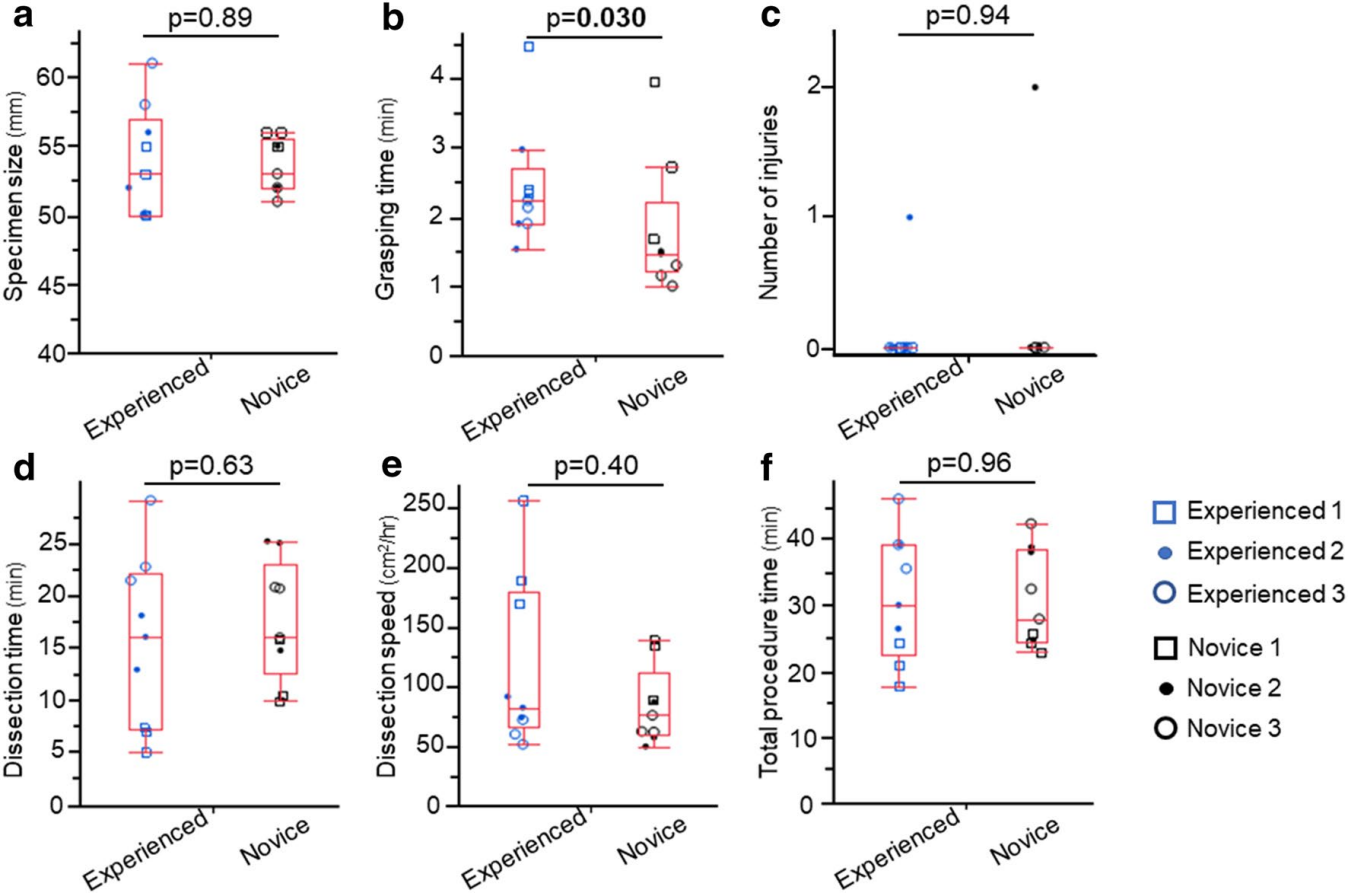
Difference in ESD procedures performed between the experienced and the novice. (**a**) Resected specimen size. (**b**) Grasping time. (**c**) Number of muscularis propria injuries. (**d**) Submucosal dissection time. (**e**) Submucosal dissection speed. (**f**) Total procedure time. Each dot indicates each procedure. The same shape means procedures performed by the same endoscopist (red dot; the experienced, blue dot; the novice).

On subgroup analysis of ESD procedures using DESP + IgE performed by the experienced (n = 9) and the novice (n = 9) endoscopists, grasping time was significantly longer in the experienced group than the novice group (experienced vs. novice = 2.2 [range 1.5–4.5] vs. 1.5 [1.0–4.0] min, *p* = 0.030, Fig. 5). On the contrary, there were no significant differences in other parameters including submucosal dissection time (experienced vs. novice = 16.1 vs. 16.0 min, *p* = 0.63), total procedure time (29.9 vs. 27.8 min, *p* = 0.96), and the number of muscularis propria injuries (experienced vs. novice = 0 [0–1] vs. 0 [0–2], *p* = 0.94).

## Discussion

This study demonstrated that the use of DESP and the flexible grasper (Ig/IgE) allow for the removal of large polyps in the right colon with a significantly shorter dissection time, total procedure time, and fewer injuries than standard ESD technique in an ex vivo porcine model. Moreover, IgE provided a broader reach of the grasper and decreased ESD procedural times and submucosal injection volumes. Finally, this surgical platform and these tools may help bridge the gap of skills in ESD procedures between the expert and the novice.

In order to access the more proximal colon, endoscopes and devices pass along the outer side of the intestinal curves and loops, leading to limited access to inner lesions, especially lesions located just beyond the splenic flexure and the hepatic flexure^17^. In this study, the Ig instrument could not reach behind each flexure. Moreover, the same difficulty also occurred in the ascending colon, the transverse colon, and the descending colon since the overtube device itself made the wide intestinal arch. The elbow bending function can resolve this issue, allowing the IgE to reach everywhere in the large intestine.

In this study, each procedure was able to successfully reach the cecum. Navigation of the colonoscope equipped with DESP may be more difficult as it required assistance with abdominal pressure, unlike standard colonoscopy. However, the simplified colon model does not allow demonstration of the advantages of the balloon technique that assists navigation under more challenging situations^18^. Similarly, the advantages of the double-balloon platform to improve the endoscope operability in the right colon were not demonstrated in this simplified model^15,19^.

Traction devices have been introduced to provide tension and counter-tension to facilitate resection of larger lesions, more complex lesions, and lesions located in difficult locations. However, few traction devices can be used anywhere in the large intestine, provide real-time dynamic traction in all directions, and move independently from the endoscope, allowing for continuous direct visualization of the target plane of submucosa. The S–O clip (Zeon Medical, Tokyo, Japan), traction wire (ProdiGI™ Traction Wire, Medtronic, Ireland) and elastic band (Elastic Traction Device; Micro-Tech Endoscopy USA Inc, Ann Arbor, Mich, USA) can be used in the right colon and provide traction in all directions, but it cannot be repositioned during the procedure^20,21^. The clip-nylon method and the suture/clip method utilizing the FB of the double-balloon interventional platform (DEIP) can provide real-time traction, but only in a single dimension, proximal and distal/back and forth^19,22,23^. Robotic platforms have not been applied to the right colon yet, though new technology might resolve this limitation^24^.

The dexterity of IgE led to significantly shorter dissection time and shorter total procedure time compared to using Ig. First, IgE was able to reach the targeted site, even the inner side at the ascending colon which was out of the Ig’s reach (Fig. 3c). Second, despite the slightly longer grasping time of IgE (Ig vs. IgE = 1.5 vs. 1.9 min), IgE could provide more traction per grasp using both elbow bending and tip articulation, leading to better visualization and reducing the need for regrasping. This novel traction method may also be particularly helpful for lesions suspected to have submucosal invasions, mucosal lesions with fibrosis, residual/recurrence lesions after incomplete endoscopic resection, and non-polypoid colorectal dysplasia with inflammatory bowel disease; these lesions are difficult to lift due to their submucosal fibrosis^14,25^.

This flexible grasper was not used during mucosal incision; in fact, there was no significant difference in mucosal incision time between the three techniques. This grasper has the potential to make mucosal incision easier by providing stability and tension, as observed with endoscopic models of robotic surgery^24^. Using the grasper during mucosal incision may further emphasize the benefits of this instrument. Moreover, the use of an additional knife/scissors in combination with IgE might also be helpful. These multi-instrument techniques should be investigated further. After tumor resection, closure of large mucosal defects (> 20 mm) in the right colon is recommended^3^. We believe the ease of mucosal closure would also greatly facilitated using the grasper to approximate the two edges of the defect^26^.

In the United States, ESD has been less widely adopted, compared to East Asian countries, since the lower incidence of gastric cancer forces endoscopists to start with the more challenging colorectal tumors, leading to the increased risk and a longer time to acquire skills in ESD. In our study, there was no difference in ESD procedural outcomes using DESP + IgE between the experienced and the novice groups. The novice could perform ESD efficiently and safely as well as the expert. This surgical platform has the potential to bridge the gap between the expert-novice differences, and shorten the learning curve of ESD.

There are several limitations in this study.

A synthetic colon, which keeps the shape compared to human colons, was used to investigate the reach of Ig/IgE. The function of Ig/IgE might be underestimated since we could collapse the colon clinically in grasping the tissue. Nevertheless, we experienced Ig wasn’t able to reach the lesion in certain locations.
(2) The ex-vivo model in this study has no peristalsis or bleeding, so performing ESD may be more difficult clinically. On the other hand, the simplicity of the model may underestimate the benefits of DESP and IgE grasper. A similar double-balloon platform, DEIP, is clinically used in the right colon and has demonstrated the benefits of the double-balloon platform in navigation. Traction devices have also been used for ESD and demonstrated clinical advantages. However, DESP, which has two more external channels, creates a larger device and its application may be limited in patients who have a narrowed colon due to diverticulitis and inflammatory bowel diseases.
(3) All lesions were 4 cm in diameter and placed at the posterior wall of the ascending colon to limit variability. The use of this traction device will need to be evaluated for resection of other challenging lesions and lesions at other challenging locations, such as circumferential lesions or lesions at the hepatic flexure or under a fold. Furthermore, this platform cannot be rotated in the proximal colon; therefore, it may be more difficult to excise the lesions located at the anterior or lateral walls.
(4) IgE may require additional time to obtain a good traction since it is necessary to articulate both distal tip and elbow-like bending section. This also highlights the need for multiple endoscopists to perform ESD and to articulate the grasper, and an assistant to secure the platform and inflate/ deflate the balloons.
(5) The procedure time may be exaggerated by the use of normal saline as the submucosal injection fluid, not a long-lasting gel. The need for repeated injections with longer procedures is exaggerated with the use of short-acting injection fluid. Additionally, the need for repeated injections further increases the total procedure time^27^.
All participants are advanced endoscopists and participants familiar with the ex-vivo research model and using this endolumenal platform. Therefore, this study didn’t investigate the learning curve of this platform^9^. Our previous study showed the experience in five ESD procedures might be enough in an ex-vivo model^16^.
This was a preclinical study. No other traction comparator was done. Larger human feasibility studies are warranted to validate these results.

In conclusion, we demonstrated a novel flexible grasper with DESP likely represents an important traction tool for removal of large pseudo-polyps in the right colon. This new technology has the potential to expand the capacity for endoluminal resection, leading to lower colectomy rates for complex polyps.

## Supplementary Information

Supplementary Video 1.

Supplementary Video 2.

## Data Availability

All data and materials are available in this study.

## Abbreviations

AB: Aft balloon
DEIP: Double-balloon interventional surgical platform
DESP: Double-balloon endolumenal surgical platform
EMR: Endoscopic mucosal resection
ESD: Endoscopic submucosal dissection
FB: Fore balloon
Ig: Endolumenal interventional grasper
IgE: Endolumenal interventional grasper with elbow
STD: Standard ESD technique
TZ: Therapeutic zone
